# Anaerobic Exercise-Induced Activation of Antioxidant Enzymes in the Blood of Women and Men

**DOI:** 10.3389/fphys.2018.01006

**Published:** 2018-07-27

**Authors:** Magdalena Wiecek, Jadwiga Szymura, Marcin Maciejczyk, Malgorzata Kantorowicz, Zbigniew Szygula

**Affiliations:** ^1^Department of Physiology and Biochemistry, Faculty of Physical Education and Sport, University of Physical Education in Krakow, Krakow, Poland; ^2^Department of Clinical Rehabilitation, Faculty of Motor Rehabilitation, University of Physical Education in Krakow, Krakow, Poland; ^3^Faculty of Physical Education and Sport, University of Physical Education in Krakow, Krakow, Poland; ^4^Department of Sports Medicine and Human Nutrition, Faculty of Physical Education and Sport, University of Physical Education in Krakow, Krakow, Poland

**Keywords:** anaerobic exercise, gender differences, enzymatic antioxidants, oxidative stress, superoxide dismutase, catalase, glutathione peroxidase

## Abstract

**Objective:** Physical exercise changes redox balance in the blood. The study aim is to determine gender-related differences in enzymatic antioxidant defense [superoxide dismutase, catalase (CAT), and glutathione peroxidase (GPx)] during the initial period following anaerobic exercise and 24 h after its completion.

**Methods:** Young, non-training participants (10 women and 10 men) performed a single anaerobic exercise, which was a 20-s maximal cycling sprint test. Blood was collected before and after completing the anaerobic exercise, i.e., after 3, 15, 30, and 60 min and after 24 h. Lactate concentration, and the superoxide dismutase, CAT, and GPx activity were determined. The results were adapted to the changes in plasma volume.

**Results:** Anaerobic exercise induced a significant increase in lactate concentration, similar among both sexes. Anaerobic exercise evokes identical changes in the activity of antioxidant enzymes in the blood plasma of women and men, which is dependent on anaerobic capacity. In the early phase of restitution, the activity of antioxidant enzymes decreases; 24 h after anaerobic exercise, GPx activity in the blood plasma of women and men is higher than before the exercise.

**Conclusion:** There are no gender-related differences concerning changes in plasma antioxidant activity after anaerobic exercise. Depending on the antioxidant enzyme, changes of activity differ in time after the end of the anaerobic exercise.

## Introduction

Redox balance is of great importance in the regulation of biochemical processes. With insufficient antioxidant defense, deregulation of intra- and extracellular processes occurs (oxidative stress) as a result of excessive production of reactive oxygen species (ROS), such as hydrogen peroxide (H_2_O_2_), superoxide anion radical (O_2_^•–^), and hydroxyl radical (HO^•^). Antioxidant defense comprises non-enzymatic and enzymatic antioxidants (Sies, 1997; Durackova, 2010).

Both O_2_^•–^ as well as HO^•^ are highly reactive free radicals due to the presence of unpaired electrons on the outer shell. As a result, they easily oxidize, among others, unsaturated fatty acids, proteins, and DNA, leading to the abnormal modification of molecules, and ultimately, to disturbances or loss of cellular function (Sies, 1997; Durackova, 2010).

Reactive oxygen species produced in low concentrations (intracellular signaling) influence the regulation and integration of biochemical processes. They activate primary signaling pathways dependent on redox status. Among others, they cause the upregulation of synthesis and activity of enzymatic antioxidants, which are responsible for the elimination of oxidants or the prevention of oxidation reactions (Ji et al., 2008). It has also been demonstrated that ROS are essential in the process of post-workout myocyte and muscle hypertrophy regeneration (Kozakowska et al., 2015).

Antioxidative enzymes are an important antioxidant defense line. They do not allow free radicals to react with macromolecules and interrupt free radical oxidation reactions. Their reactions are connected with one another. The main function of the enzyme antioxidant system is performed by superoxide dismutases: cytoplasmic – Cu/ZnSOD, mitochondrial – MnSOD, and extracellular – EC-SOD (also containing Cu/Zn), which catalyze the O_2_^•–^ dismutation reaction to H_2_O_2_ and molecular oxygen. H_2_O_2_ is detoxified in catalase (CAT) and glutathione peroxidase (GPx) reactions. The product of the H_2_O_2_ disproportionation reaction catalyzed by CAT is molecular oxygen and water. GPx leads to the reduction of inorganic (H_2_O_2_) and organic peroxides (ROOH) along with glutathione (GSH). However, if the activity/concentration of these enzymes is not sufficient, H_2_O_2_ (in the Fenton reaction) is transformed into a more reactive HO^•^ (Galecka et al., 2008; Durackova, 2010).

Under physiological conditions, the source of ROS is the metabolic processes (aerobic and/or anaerobic) associated with ATP resynthesis, which undergo increased activation during physical exercise. ROS arise in the internal mitochondrial membrane during oxidative phosphorylation, as well as in the NAD(P)H oxidase and xanthine oxidase (XO) reactions and as a result of catecholamine autooxidation (Morales-Alamo and Calbet, 2014; He et al., 2016).

In our earlier research, we demonstrated that exercises at supramaximal (Wiecek et al., 2015b, 2017a), maximal (Wiecek et al., 2015a, 2017b), and submaximal intensities (Wiecek et al., 2017c) have influence on redox balance in the blood of women and men, and their effects are different depending on the intensity and duration of the effort as well as the type of engaged muscle work.

Previous studies have indicated greater antioxidant capacity in women. It has been shown that the effect of estrogens on the estrogenic receptor is upregulation of mitogen-activated protein kinases (MAPK) and subsequent activation of the NF-κB signaling pathway, which consequently results in increased gene expression for MnSOD and GPx (Vina et al., 2000).

Other authors have found that after anaerobic exercise performed by men, the activity of the NF-κB increases in the nuclei of peripheral blood mononuclear cells under the influence of ROS, which affects the upregulation of gene expression of antioxidant enzymes increasing total antioxidant capacity (TAC) of the blood (Cuevas et al., 2005). At the same time, in response to a single anaerobic effort, there were no gender-related differences in the activation of intracellular signaling pathways associated, among others, with redox balance (Fuentes et al., 2012).

The results of studies on enzymatic antioxidant defense after efforts are ambiguous. Following graded exercise, a slightly higher activity of GPx was noted in women than men. There were no differences in the level of lipid oxidation between women and men (Rush and Sandiford, 2003), while a significant increase in SOD activity was only observed in women (Akkus, 2011). Karabulut (2011), comparing the post-anaerobic exercise results for women and men, noted a significant increase in malondialdehyde concentration and a decrease in GSH concentration, demonstrating the occurrence of oxidative stress in men, in contrast to women, for whom significant changes in the range of these markers were not determined. In turn, our earlier research showed that anaerobic exercise results in similar blood changes regarding oxidative stress index, TAC, and non-enzymatic antioxidants for both sexes (Wiecek et al., 2015b). In contrast, activation of XO was significantly higher in women than men (Wiecek et al., 2017a). It was also noted that larger content of type II fibers and peak anaerobic power positively correlated with the level of post-exercise oxidative stress (Quindry et al., 2011). In our research, we stated that anaerobic exercise causes a smaller increase in XO activity in people with higher anaerobic performance (Wiecek et al., 2017a). At the same time, it is known that men generate greater anaerobic power and have higher anaerobic capacity than women (Wiecek et al., 2015b, 2016).

For many years, research has been focused on blood prooxidant and antioxidant balance during and after physical exercise, rarely relating to gender differences (Malorni et al., 2007; Powers and Jackson, 2008; Morales-Alamo and Calbet, 2014; Jackson et al., 2016).

The aim of our research was to evaluate the effect of a single anaerobic exercise on the activity of antioxidant enzymes and to determine whether there are any gender-related differences in enzymatic antioxidant defense during the early period following anaerobic exercise (of supramaximal intensity) and 24 h after its completion. We put forward the hypothesis that anaerobic exercise induces greater changes in antioxidant enzyme activity in the blood of women compared to men.

In order to verify the hypothesis, we determined changes in the activity of SOD, CAT, and GPx in the blood plasma of men and women several times during the first hour following a single anaerobic exercise and 24 h after its completion.

## Materials and Methods

### Participants

Ten women and 10 men aged 19–24 comprised the study participants. They were healthy, non-smokers, non-training but physically active individuals (light to moderate exercise, three times a week). Women were characterized by a normal two-phase menstrual cycle (27–32 days) and did not take any hormonal preparations, including oral contraceptives.

### Study Design

The study was carried out in accordance with the principles of the Declaration of Helsinki. The research procedures were approved by the Bioethical Commission of the Regional Medical Chamber (opinion No. 81/KBL/OIL/2013). Participation in the research was voluntary. The participants were informed about its purpose and were familiarized with the laboratory procedures. They expressed written consent to participate in the trial.

On the basis of medical qualifications (medical history, physical examination, and ECG analysis), no medical contraindications to perform anaerobic efforts by participants were found.

Exercise tests were performed under the supervision of a sports medicine physician at 9.00–11.00 in the morning, in thermo-neutral conditions. Women performed the anaerobic test between days 6 and 9 of the menstrual cycle (half of the follicular phase). For 2 days before, on the day of and 24 h after the test, the participants did not perform any intense physical efforts or use a sauna, cryotherapy, or other biological renewal treatments, they did not consume caffeine-containing products or other stimulants.

None of the participants were on a vegetarian or vegan diet. No one used antioxidative supplements. Additionally, during the 7 days preceding the exercise test, participants applied a standardized diet (designed by a dietician) in terms of the percentage of consumed protein (15%), fat (30%), and carbohydrates (55%) in meeting energy needs (2,800 kcal/day for men and 2,000 kcal/day for women). Dosages of C, and E antioxidant vitamin, and β-carotene were: 75 mg/day, 10 mg/day, and 630 μg equivalent of retinol/day, respectively. The subjects performed an anaerobic effort after an at least 8-h night rest and about 2 h after a standard meal that was prepared according to the dietician’s guidelines. The participants did not drink any fluids immediately before the exercise or during restitution.

### Somatic Measurements

Body height (Martin type anthropometer), total body mass (BM), percentage fat content, and lean body mass (LBM) were measured (Jawon IOI-353 Body Composite Analyzer, Korea). Body mass index (BMI) was calculated for each participant (**Table 1**).

**Table 1:** Age and somatic features of the participants.

	Age years	BH cm	BM kg	LBM kg	BF %	BMI kg/m^2^
Women	22.0 ± 0.5	166.6 ± 1.1	59.8 ± 2.1	45.2 ± 1.3	24.2 ± 0.6	21.5 ± 0.6
Men	21.6 ± 0.4	180.1 ± 1.7	77.1 ± 2.7	63.0 ± 2.3	18.4 ± 0.9	23.7 ± 0.5
*p*-value	0.54	<0.01	<0.01	<0.01	<0.01	<0.01

### Anaerobic Effort – Cycle Sprint

The stress test consisted of three phases: warm-up (4 min), rest (4 min), and cycle sprint (20 s). The test was carried out in accordance with the earlier published methodology (Wiecek et al., 2015b).

The cycle sprint was performed with a load totaling 7.5% of BM for men and 6.5% of BM for women. This trial was based on obtaining the maximal pedaling cadence in the shortest possible time and then maintaining it for 20 s. The time of individual revolutions was measured with an accuracy of 1 ms (magnetic meter) and the maximal and mean power obtained as well as total work performed during the test were automatically calculated (MCE software, JBA Staniak, Poland), which was presented relative to the BM and LBM of the participants.

### Blood Collection and Biochemical Assay

Venous blood was collected before beginning the warm-up – baseline and five times after completing the anaerobic exercise, i.e., after 3, 15, 30, and 60 min and after 24 h. Cannulas, catheters, and disposable syringes with physiological saline solution and a vacuum tube system (Vacutainer BD, United States) were used. During blood collection, the participant was in a seated position. About 15 min before the warm-up, a cannula was inserted into the venous ulnar vessels, which was secured against clotting by rinsing it with saline solution after application and each time before and after blood collection (1 ml of 0.9% NaCl was injected). After taking the blood, the cannula was closed with a catheter. Each time, blood was put into tubes containing EDTA. The first tube (2 ml) with blood was discarded.

Hemoglobin (HGB) concentration and hematocrit (HCT) were determined immediately in the whole blood from the second test-tube (2 ml), and then, the blood was stored on ice and centrifuged as soon as possible for 15 min at 4°C at the RCF of 1,000 × g (MPW 351R, Poland). The blood plasma samples were stored until analysis at -80°C (ULF 390 Arctiko, Denmark). HGB concentration was determined by spectrophotometry (540 nm) with the cyanide-methemoglobin method using Drabkin’s reagent (Poland). HCT was determined using the micro-HCT method in triplicate and the mean results were calculated.

In the blood plasma, using spectrophotometry (Infinite M200 PRO TECAN, Grödig, Austria), superoxide dismutase activity (SOD, EC 1.15.1.1), CAT (EC 1.11.1.6), and GPx (EC 1.11.1.9) were determined according to the procedure specified by the manufacturer of the Cayman Chemical Company (United States), Superoxide Dismutase Assay Kit 706002, Catalase Assay Kit 707002, and Glutathione Peroxidase Assay Kit 703102 reagent kits, respectively. The CV intra- and inter-assay for the tests was 3.2 and 3.7%, 3.8 and 9.9%, 5.7 and 7.2%, respectively, for the SOD, CAT, and GPx assay kits.

The SOD assay uses tetrazolium salt for detection of superoxide anions generated by xanthine and hypoxanthine. One unit of SOD is defined as the amount of enzymes needed to exhibit 50% of dismutation of the superoxide anions (U/ml). SOD activity is standardized using the cytochrom c and XO coupled assay. Absorbance is measured at 450 nm.

The method for determining CAT activity is based on the methanol oxidation reaction in the presence of an optimal H_2_O_2_ concentration. The generated formaldehyde forms a colored product from 4-amino-3-hydrazino-5-mercapto-1,2,4-triazole (Purpald), the concentration of which is measured using spectrophotometry (540 nm). One unit of CAT activity is defined as the amount of enzymes causing the formation of 1 nmol of formaldehyde per minute (nmol/min/ml).

GPx activity is measured indirectly. Oxidized glutathione, produced in a reaction catalyzed by GPx, is reduced by glutathione reductase and NADPH. In this reaction, NADPH oxidation to NADP^+^ is accompanied by a decrease in absorbance at 340 nm, which is also proportional to the GPx activity in the sample. One unit of GPx activity corresponds to the amount of the enzyme causing the oxidation of 1 nmol NADPH to NADP^+^ per minute (nmol/min/ml).

Lactate concentration was determined in the capillary blood plasma (from the finger tip) before and 3 min after the exercise. Test-tubes (300 μl) with EDTA and sodium fluoride (glycolysis inhibitor) were used. The blood was immediately centrifuged for 3 min at an RCF of 14,300 × g (MPW 55 centrifuge, Poland) and lactate concentration was determined with the enzymatic method (Randox L-Lactate assay, United Kingdom) using spectrophotometry (550 nm). The assay sensitivity amounted to 0.165 mmol/l, and its linearity was up to 19.7 mmol/l.

The results of SOD, CAT, and GPX and lactate concentration were corrected by the percentage changes in plasma volume (%ΔPV) according to the Kraemer and Brown formula (Kraemer and Brown, 1986). %ΔPV was calculated on the basis of changes in HGB concentration (g/dl) and HCT values (%) (Dill and Costill, 1974; Harrison et al., 1982).

$$\begin{array}{c} W\;=\;(\%\Delta \mathrm{PV}\;\times \;0.01\;\times \;W_{\mathrm{after}})\;+\;W_{\mathrm{after}} \end{array}$$

where

W – corrected level of the indicator and W_after_ – indicator level measured after exercise (Kraemer and Brown, 1986).

%ΔPV = 100 × {(HGB_before_ / HGB_after_) × [100 - (0.874 × HCT_after_)]/[100 - (0.874 × HCT_before_)] - 1}

where

HGB_before_ and HGB_after_ – HGB concentration before and after exercise, respectively;
HCT_before_ and HCT_after_ – HCT concentration before and after exercise, respectively (Dill and Costill, 1974; Harrison et al., 1982).

### Statistical Analysis

All statistical analyses were performed using Statistica 10 (Stat-Soft, Inc., United States). The charts were also created via this program. Data are presented as means ± standard error (*SE*). The data distribution was checked using the Shapiro–Wilk test. Significance of gender-related differences for single measurements was checked using the *t*-test for independent samples. Two-way analysis of variance with repeated measurements was performed to compare differences for genders among post-exercise changes in SOD, CAT, and GPx activity. In case of significant influence of the main factor (sex, anaerobic effort, or sex–anaerobic effort interaction), the significance of differences between specific averages was checked using analysis of planned comparisons. It was checked whether there was a correlation (Pearson’s test) between the activity of SOD, CAT, and GPx in the plasma, measured at particular time points, and statistically significant post-exercise changes in antioxidant enzyme activity, as well as the total work performed during the test and the maximal and average power obtained in the test, which were expressed relative to BM. For all variables, the mean differences at the level of *p* < 0.05 were considered statistically significant.

## Results

### Anaerobic Capacity

The total work performed by men during anaerobic exercise (188.1 ± 3.9 J/kgBM and 230.5 ± 4.9 J/kgLBM) was significantly greater (*p* < 0.01) than the work performed by women (146.5 ± 3.1 J/kgBM and 193.7 ± 3.9 J/kgLBM). During anaerobic exercise, the men generated significantly higher (*p* < 0.01) maximal power (11.3 ± 0.3 W/kgBM and 13.8 ± 0.3 W/kgLBM) and achieved higher (*p* < 0.01) mean power (9.4 ± 0.2 W/kgBM and 11.5 ± 0.2 W/kgLBM) than the women, for which the results were 8.6 ± 0.1 W/kgBM (11.3 ± 0.2 W/kgLBM) and 7.3 ± 0.2 W/kgBM (9.7 ± 0.2 W/kgLBM), respectively.

### Lactate Concentration

Before the exercise, the lactate concentration in women totaled 1.00 ± 0.14 mmol/l (*p* > 0.05) and was comparable to the level in men (1.06 ± 0.12 mmol/l). Anaerobic exercise induced a significant increase in lactate concentration among both sexes, by 6.83 ± 0.62 mmol/l (*p* < 0.01) and 7.97 ± 0.50 mmol/l (*p* < 0.01) in women and men, respectively. The increases in lactate concentration were similar in both sexes (sex/exercise effect *p* > 0.05).

### Antioxidant Enzymes’ Activity

**FIGURE 1:**
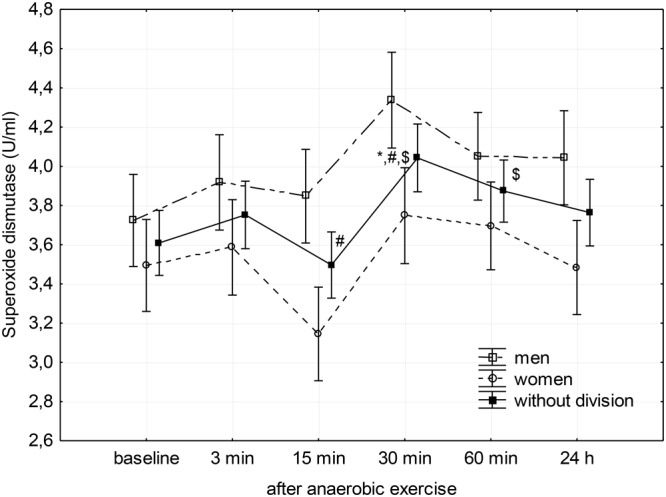
Baseline and changes in superoxide dismutase activity in blood plasma after anaerobic exercise. Data are presented as mean ± *SE*; statistically significant differences: ^∗^vs. baseline, ^#^vs. 3 min, and ^$^vs. 15 min after anaerobic exercise (*p* < 0.05).

**FIGURE 2:**
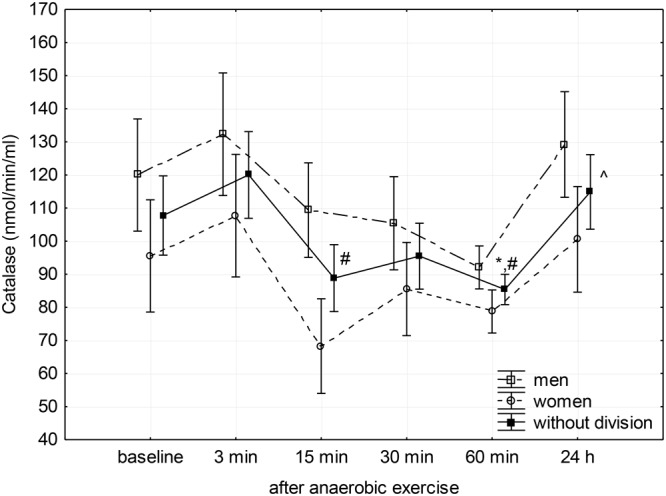
Baseline and changes in catalase activity in blood plasma after anaerobic exercise. Data are presented as mean ±*SE*; statistically significant differences: ^∗^vs. baseline, ^#^vs. 3 min, and ^∧^vs. 60 min after anaerobic exercise (*p* < 0.05).

**FIGURE 3:**
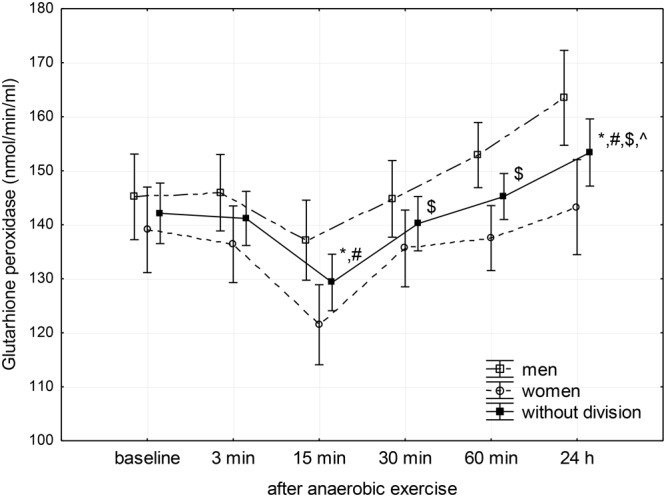
Baseline and changes in glutathione peroxidase activity in blood plasma after anaerobic exercise. Data are presented as mean ± *SE*; statistically significant differences: ^∗^vs. baseline, ^#^vs. 3 min, ^$^vs. 15 min, and ^∧^vs. 30 min after anaerobic exercise (*p* < 0.05).

Changes in SOD, CAT, and GPx activity in the blood plasma after anaerobic exercise are presented graphically (**Figures 1–3**). After anaerobic exercise, there were significant changes in the activity of SOD, CAT, and GPx in the blood plasma (main factor: anaerobic exercise, *p* < 0.05), which were similar in the women and men (sex–anaerobic effort interaction; *p* > 0.05). The activity of SOD, CAT, and GPx was similar in women and men (main factor: sex) in all the measurements (*p* > 0.05). The detailed results of statistical analysis for the impact of major factors are shown in **Table 2**.

**Table 2:** Assessment of sex, anaerobic exercise, and sex–anaerobic exercise interaction influence on antioxidant enzyme activity in the blood plasma based on the results of two-way analysis of variance with repeated measures.

Variable	Sex	Anaerobic exercise	Sex–anaerobic exercise interaction
		***p*-value (*F*)**	
SOD	0.10 (2.93)	0.01 (3.36)	0.59 (0.75)
CAT	0.07 (3.61)	0.03 (2.62)	0.92 (0.28)
GPx	0.17 (2.07)	<0.01 (6.60)	0.59 (0.75)

*SOD, superoxide dismutase; CAT, catalase; GPx, glutathione peroxidase; statistical power (1-β) and size effect (η^*2*^) to assess the effect of anaerobic exercise on the activity of antioxidant enzymes at *p* = 0.05 were, respectively, 0.89 and 0.16 for SOD, 0.78 and 0.13 for CAT, and 0.99 and 0.27 for GPx.*

*Post hoc* analysis of the main factor – anaerobic effort (without division according to gender) – showed that SOD activity in the third minute following completion of anaerobic exercise was close to the baseline value, after which it significantly reduced during the 15th minute of rest (*p* = 0.04). Then, it significantly increased reaching a value significantly higher than that measured before exercise in the 30th minute (*p* = 0.02), as well as the value measured at the 3rd (*p* = 0.03) and 15th minutes (*p* < 0.01) after its completion. SOD activity remained elevated for another 30 min (*p* < 0.01); 24 h after the end of the anaerobic exercise, there was no significant difference in the activity of SOD in the blood plasma compared to baseline (**Figure 1**).

Catalase activity decreased significantly (*p* = 0.01) between the 3rd and 15th minute after anaerobic exercise, reaching a significantly lower level than the baseline during the 60th after completion (*p* = 0.05) and measured during the 3rd minute (*p* = 0.01) following exercise. The CAT activity then increased to a value similar to baseline 24 h after the anaerobic effort (**Figure 2**).

Glutathione peroxidase activity showed a significant decrease below baseline level between the 3rd and 15th minute after anaerobic exercise, reaching a value lower than both the baseline (*p* < 0.01) and the value recorded during the 3rd minute following exercise (*p* < 0.01). Next, there was a significant linear increase in GPx activity reaching a value higher than baseline (*p* = 0.04) 24 h after the anaerobic exercise (**Figure 3**).

### Correlations

A significant (*p* < 0.05) positive correlation (*r* = 0.49) between the decrease in SOD and CAT activity was found in the 15th minute after anaerobic exercise.

There was a significant (*p* < 0.05) positive relationship between SOD activity in the plasma during the 30th minute after the completion of anaerobic exercise, as well as an increase in GPx activity occurring 24 h after exercise, and relative to BM: total work (J/kgBM) performed during anaerobic exercise (*r* = 0.55 and *r* = 0.50, respectively) and maximal (*r* = 0.54 and *r* = 0.45, respectively) and mean power (W/kgBM) generated in the test (*r* = 0.54 and *r* = 0.51, respectively). When the results of the anaerobic test were expressed in relation to LBM, it was stated that a significant (*p* < 0.05) positive correlation can be observed between SOD in the 30th minute following the effort and total work (*r* = 0.51), maximal (*r* = 0.54), and mean power (*r* = 0.51). A significant positive correlation (*p* < 0.05) was also noted between the increase in GPx activity occurring 24 h after exercise, total work (J/kgLBM), and mean anaerobic power (W/kgLBM); *r* = 0.50 as well as *r* = 0.49, respectively. However, there was no significant correlation between the increase in GPx activity and maximal anaerobic power (W/kgLBM).

## Discussion

Our research has shown that a single anaerobic exercise induces significant changes in the activity of antioxidant enzymes in the blood plasma. Depending on the enzyme, these changes differ in time after the end of the anaerobic exercise. Simultaneously, we have shown that in young physically active people, the resting level and exercise response of enzymatic antioxidant defense is similar regardless of gender; 24 h after the completion of anaerobic exercise, the activity of GPx in the blood of women and men was increased.

Research by Baghaiee et al. (2016), which compared the resting gene expression in lymphocytes for antioxidant enzyme proteins in women and in men, showed higher mRNA expression for MnSOD in women, but without significant gender-related differences in gene expression for Cu/ZnSOD and CAT. Total plasma antioxidant capacity was higher in women (Baghaiee et al., 2016).

In our trial, we did not define gene expression but compared the activity of antioxidant enzymes in women and men. Enzyme activity can change without altering gene expression, and vice versa (Fisher et al., 2011). We did not find any significant gender-related differences in the activity of SOD, CAT, and GPx in the blood plasma prior to exercise. It should be noted that in our research, we determined the activity of all forms of SOD simultaneously (total-SOD). The results of previous studies are varied. For example, higher levels of MnSOD activity in lymphocytes (Sureda et al., 2008), CAT in blood plasma (Pepe et al., 2009), and GPx in erythrocytes (Kowalska and Milnerowicz, 2016) were found in women. In other studies, as in ours, there were no gender-dependent differences in the activity of GPx (Kowalska and Milnerowicz, 2016) and total-SOD (Karabulut, 2011) in the blood plasma or Cu/ZnSOD in erythrocytes (Kowalska and Milnerowicz, 2016). In men, there was also higher CAT, Cu/ZnSOD (Kowalska and Milnerowicz, 2016) and total-SOD activity in the blood plasma (Pepe et al., 2009), as well as greater GPx activity in lymphocytes (Ferrer et al., 2009). In the group of young individuals practicing various sports disciplines, despite the higher total-SOD activity in the blood plasma of women (73 people), the level of other biochemical indicators showed the predominance of oxidation processes in women compared to men (65 people; Dopsaj et al., 2011).

The level of sex hormones in women does not significantly affect the maximal or average anaerobic power (Wiecek et al., 2016; Pestana et al., 2017). There are also studies in which correlations between the level of estradiol and oxidative stress markers have not yet been confirmed, nor were there any differences in the total-SOD and EC-SOD activity during the menstrual cycle in young women (Joo et al., 2004). However, research has shown that in women with normal biphasic menstrual cycles, estradiol levels negatively correlate with post-exercise (30 min, 60%VO_2_max) changes (decrease) in total-SOD and EC-SOD activity, and free radicals produced during exercise metabolism are more easily eliminated when the estradiol level is higher (Joo et al., 2004). In these studies, no correlations were found between the level of progesterone and the level of free radicals, or the activity of antioxidant enzymes (Joo et al., 2004). In our research, to avoid the potential effects of different estradiol concentrations in the course of the menstrual cycle on post-workout changes in antioxidant enzymes, all women performed anaerobic exercise during the follicular phase.

The 30-s Wingate test (WAnT), called the anaerobic effort (Bar-Or, 1987), is a common exercise test used to assess maximum power and anaerobic capacity. However, energy in such an effort is mixed. According to Beneke et al. (2002), during the WAnT, fractions of the energy from aerobics, anaerobic alactic, and lactic acid metabolism total about 18.6, 31.1, and 50.3%, respectively. Other authors estimate the contribution of oxygen sources in such a test to be 16%, while stating that the maximal power of the glycolytic system occurs at the end of the first 15 s of an effort, then decreases, and the share of aerobic energy increases (Smith and Hill, 1991). In our research, we used a 20-s cycle-sprint as an anaerobic effort. It is a reliable and validated leg anaerobic power test in humans and may replace the classic WAnT (Bar-Or, 1987; Attia et al., 2014). At the same time, shortening the duration of the effort causes the share of aerobic energy to decrease (Smith and Hill, 1991). Under these conditions, the main source of ROS are reactions catalyzed by NADPH oxidase, XO, and catecholamine autooxidation; nonetheless, the mitochondrial source of ROS cannot be completely excluded (Morales-Alamo and Calbet, 2014; He et al., 2016).

According to previous studies, depending on the measured prooxidant and antioxidant balance indicators, their changes can occur at different times after physical exercise (Michailidis et al., 2007; Wiecek et al., 2015b). Also in our research, we found that to observe the changes in the activity of individual antioxidant enzymes, the time of collecting biological material after completing the effort is important. The first significant changes in the activity of antioxidant enzymes were found 15 min after the completion of the anaerobic exercise and were manifested by a decrease in the activity of SOD, CAT, and GPx in the blood plasma at a similar intensity in both women and men. In the further phase of restitution, the course of activity changes was different for individual antioxidant enzymes, but still similar in both sexes. In contrast to the research by Wiecek et al. (2015b), with the participation of the same volunteers, which showed a significant increase in TAC in the blood and an increase in non-enzymatic antioxidants (β-carotene and α-tocopherol) in the first 3 min after 20 s of anaerobic exercise, we did not, at the same time, find any changes in the enzyme antioxidant activity. The noted decrease in SOD activity may be the result of the reaction being inhibited by H_2_O_2_ formed in a high concentration, which may additionally be supported by post-exercise acid-base disorder (reduction of HCO_3_^-^ concentration; Skrzycki and Czeczot, 2004; Wiecek et al., 2017a). The synergy of antioxidant enzymes (Durackova, 2010) confirms the correlation we found between the decrease in SOD and CAT activity during the 15th minute following anaerobic exercise.

Comparing post-exercise reactions in the range of enzymatic antioxidant defense in young women and men, there was no change in SOD activity after a 20-m shuttle run (Karabulut, 2011). After the gradual cycling exercise performed by young physically active people, a significant increase in CAT gene expression was found in the lymphocytes of the males, both directly after exercise and three hours after its completion, which resulted in increased TAC in the blood plasma. In the group of active women, there was an increase in TAC in the blood plasma and activation of the CAT gene expression in lymphocytes only directly after the graded exercise, while the level of mRNA for MnSOD in lymphocytes raised as late as 3 h after exercise (Baghaiee et al., 2016). Similar in women and men, and in our study, the decreases of SOD and CAT activity in the blood plasma, respectively, after 800 and 1,500 m races at 10 km/h, were reported by Pepe et al. (2009). In contrast, these changes were noted immediately after the end of the effort (Pepe et al., 2009).

In the studies by Groussard et al. (2003), men, at an age similar to our participants, performed the WAnT. There was a significant reduction in SOD activity immediately after exercise, followed by a gradual return to resting values over the next 40 min. In these trials, however, no significant changes in GPx activity were found, contrary to our results. Nonetheless, attention was drawn to the large variation of results in the study group (Groussard et al., 2003). In the research by Berzosa et al. (2011), immediately after completing the exercise performed at the intensity of VO_2_max until exhaustion, a significant increase in the activity of SOD, CAT, and GPx in the plasma of men was found. After anaerobic effort also performed by men, there was an increase in TAC and CAT activity in the blood serum during the 30th minute of restitution, and these values also increased 24 h after the exercise. GPx activity, similarly as in our studies, did not increase significantly until 24 h after the exercise, and similarly to TAC, it remained at an elevated level also after the second day of restitution (Bogdanis et al., 2013).

The changes in GPx activity that we have found are adequate to the changes in GSH and GSSG concentrations demonstrated by us after the same effort with the participation of the same individuals (Wiecek et al., 2015b). The increase in GPx activity increases the rate of GSH oxidation to GSSG with the participation of H_2_O_2_, which results in a decrease of GSH concentration, an increase in GSSG concentration, and a reduction in GSH/GSSG ratio, which was previously demonstrated (Wiecek et al., 2015b). Significant changes in the concentration of both forms of glutathione and the GSH/GSSG ratio were also observed 24 h after the end of exercise (Wiecek et al., 2015b). Furthermore, 24 h after completion of the 20-s anaerobic exercise, a significant increase in XO activity (Wiecek et al., 2017a) was found, although significant gender-dependent differences were found in the enzyme activity range. The increase in XO activity 24 h after exercise was higher in women than men. The product of a reaction catalyzed by XO is O_2_^•–^, and then H_2_O_2_ (Morales-Alamo and Calbet, 2014). GPx is responsible for the catabolism of most H_2_O_2_ produced in cells, due to the greater affinity with this substrate than CAT (Scibor and Czeczot, 2006).

In our research, the increase in GPx activity was positively correlated with the maximal and mean power generated during the 20-s bicycle sprint, as well as with the total work done in the test. These results indicate the positive effect of improved anaerobic capacity on blood antioxidant capacity. Previously, it has been shown that the TAC of the blood, which is also influenced by the activity of antioxidant enzymes, depends on physical activity (Wiecek et al., 2017d). Although the men in our study demonstrated greater anaerobic capacity, we did not find significant differences between sexes in post-exercise changes regarding antioxidative enzyme activity. It is possible that this is due to similar physical activity in both groups. The studied groups were not numerous, which may additionally influence the outcome of result statistical analysis.

### Limitation of the Study

The number of examined participants was not large. To increase the reliability of the results (the power of statistical tests), it would be necessary to increase the number of participants. We studied non-training individuals. Due to the dependence of changes in the activity of antioxidant enzymes after anaerobic exercise on anaerobic capacity, future research should also include people with high anaerobic capacity, taking changes in estradiol levels during the menstrual cycle of women into account. Furthermore, the subject of study should include both antioxidant enzymes, e.g., in erythrocytes or lymphocytes, as well as gene expression.

## Conclusion

Anaerobic exercise induces changes in the activity of antioxidant enzymes (SOD, CAT, and GPx) in the blood plasma. There are no gender-related differences in changes regarding plasma antioxidant activity after anaerobic exercise. Depending on the antioxidant enzyme, changes of activity differ in time after the end of the anaerobic exercise. In the early phase of restitution, the activity of antioxidant enzymes decreases; 24 h after the anaerobic exercise, GPx activity in the blood plasma of women and men is higher than before the effort.

## Author Contributions

MW conceived the project, procured the project funding, performed the statistical analysis, interpretation of results and drafted the final version of the manuscript. MW, JS, MM, and ZS contributed to the collection of data and reagents. MK developed and analyzed diets. All authors contributed to revising the manuscript and expressed their approval of the final submitted version.

## Conflict of Interest Statement

The authors declare that the research was conducted in the absence of any commercial or financial relationships that could be construed as a potential conflict of interest.
